# Single cell dynamics determine strength of chaos in collective network dynamics

**DOI:** 10.1186/1471-2202-12-S1-P225

**Published:** 2011-07-18

**Authors:** Michael Monteforte, Fred Wolf

**Affiliations:** 1 Max-Planck-Institute for Dynamics and Self-Organization, 37073 Goettingen, Germany; 2 BCCN, BFNT and Georg August University of Goettingen, 37073 Goettingen, Germany

## 

Cortical neurons have been found to exhibit a much higher action potential (AP) onset rapidness than expected from standard biophysical neuron models [1]. This has raised fundamental physiological questions about the origin of this phenomenon [1,2]. An important issue for the understanding of information processing in the cortex is the impact of rapid AP initiation on the collective dynamics of cortical networks. Here, we report that it in fact strongly reduces the information loss in chaotic cortical networks.

As a model of cortical networks, we analyzed spiking neuron networks in the balanced state [3]. The balanced state provides an explanation of the temporally irregular activity of cortical networks observed *in vivo*[4]. In this state neurons are driven by large input fluctuations, resulting from a dynamical balance of excitation and inhibition.

Networks of theta neurons in the balanced state exhibit strongly chaotic dynamics [5]. We recently performed an exact analysis of the full spectra of Lyapunov exponents in such networks, revealing that deterministic chaos is extensive and information is lost at strikingly high rates of up to 1 bit per spike per neuron. The theta neuron model, however, shares the relatively low AP onset rapidness of other biophysical standard neuron models.

**Figure 1:**
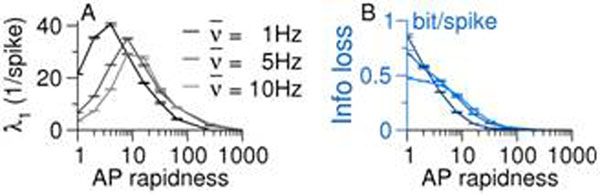
Largest Lyapunov exponent (**A**) and rate of information loss (**B**) versus AP onset rapidness in balanced networks of rapid theta neurons at three different average firing rates 1,5 and 10Hz.

Here we show that increasing the AP onset rapidness of single neurons strongly reduces the intensity of chaos in balanced networks. Based on the theta neuron model, we developed a new neuron model with variable AP onset rapidness, called the rapid theta neuron model. Parametrically increasing the AP onset rapidness in the neurons reduced the information loss in the chaotic network dynamics and could even induce a transition to stable irregular dynamics (Fig. 1).

These results reveal that the action potential rapidness of single neurons plays an important role in the collective dynamics of cortical networks. A rapid AP initiation reduces the information loss due to the chaotic dynamics. Our results thus suggest that cortical neurons may have evolved their rapid AP initiation in order to reduce the information loss in chaotic cortical networks and tune the network dynamics towards the edge of chaos.
